# Automated discovery and phylogenetic analysis of new toxin-antitoxin systems

**DOI:** 10.1186/1471-2180-8-104

**Published:** 2008-06-25

**Authors:** Julien Guglielmini, Cédric Szpirer, Michel C Milinkovitch

**Affiliations:** 1 Laboratory of Evolutionary Genetics, Institute for Molecular Biology & Medicine, Université Libre de Bruxelles (ULB), 12 rue Jeener & Brachet, 6041 Gosselies, Belgium

## Abstract

**Background:**

Although often viewed as elements "at the service of" bacteria, plasmids exhibit replication and maintenance mechanisms that make them purely "selfish DNA" candidates. Toxin-antitoxin (TA) systems are a spectacular example of such mechanisms: a gene coding for a cytotoxic stable protein is preceded by a gene coding for an unstable antitoxin. The toxin being more stable than the antitoxin, absence of the operon causes a reduction of the amount of the latter relative to the amount of the former. Thus, a cell exhibiting a TA system on a plasmid is 'condemned' either not to loose it or to die.

**Results:**

Different TA systems have been described and classified in several families, according to similarity and functional parameters. However, given the small size and large divergence among TA system sequences, it is likely that many TA systems are not annotated as such in the rapidly accumulating NCBI database. To detect these putative TA systems, we developed an algorithm that searches public databases on the basis of predefined similarity and TA-specific structural constraints. This approach, using a single starting query sequence for each of the ParE, Doc, and VapC families, and two starting sequences for the MazF/CcdB family, identified over 1,500 putative TA systems. These groups of sequences were analyzed phylogenetically for a better classification and understanding of TA systems evolution.

**Conclusion:**

The phylogenetic distributions of the newly uncovered TA systems are very different within the investigated families. The resulting phylogenetic trees are available for browsing and searching through a java program available at .

## Background

Plasmids are autonomously-replicating extra-chromosomal circular DNA molecules usually nonessential for cell survival under non-selective conditions and widely distributed in prokaryotic cells. Because plasmids sometimes bear genes that provide bacteria with functions (such as virulence, resistance to drugs, the ability to exploit a specific source of carbon) that can be adaptive in variable environments, they are often viewed as elements "at the service of" a (intra- or inter-specific) pool of bacteria, thus allowing the long-term survival of these lines or species. However, their ability to autonomously replicate makes plasmids possible purely "selfish DNA" candidates. Indeed, some plasmids exhibit features that seem to be strictly restricted to mechanisms related to their maintenance in cell lines (through replication and partitioning mechanisms) or dispersal across cell lines or species (through conjugation mechanisms).

**Figure 1:**
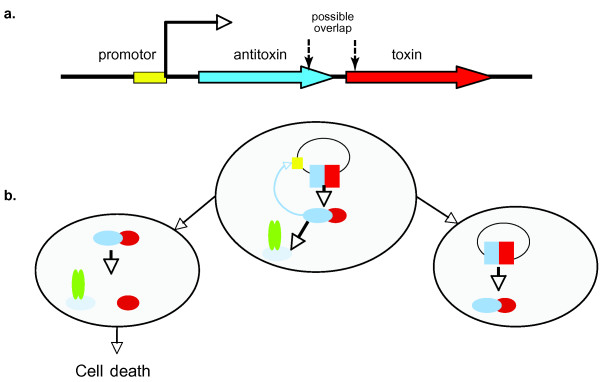
***(a) ***Organization of a canonical TA system (after [49]). ***(b) ***In the presence of the operon (mother cell and right daughter cell), the antitoxin (blue) is produced together with the poison (red) and the former neutralizes the latter; the transcription of the system is auto-regulated by the binding of the antitoxin (and/or the [antitoxin+toxin] complex) to the promoter region (yellow) of the operon. In the absence of the TA operon, the unstable antitoxin is degraded by a protease (green) and the poison can exert its toxicity on an essential intracellular host target.

Poison-antidote, or Toxin-antitoxin (TA) systems, were first discovered on plasmids [1] and associated to plasmid-maintenance mechanisms. Later, several TA systems were identified on chromosomes [2-5] and interpreted as bacterial programmed cell death or stress response loci [2,6,7]. Thought to occur in many Prokaryotic species [8], most TA systems are organized as follows: a gene coding for a cytotoxic and stable protein is preceded by a DNA sequence coding for an unstable antitoxin (or "antidote") protein or antisense RNA that can neutralize the toxic protein or its corresponding mRNA, respectively. The instability of the antitoxin protein is due to specific degradation by a protease [9,10]. Most systems investigated to date *(i) *involve a proteic (rather than RNA) antitoxin that efficiently sequesters the toxin, and *(ii) *exhibit a tandem organization with a single promoter and a small distance (about 20–30 bases), or even an overlap (about 20–30 bases), between the antitoxin and toxin genes (Fig. 1a). The transcription of the system is auto-regulated by the binding of the antitoxin (and/or the [antitoxin+toxin] complex) to the promoter region of the operon [11,12]. We hypothesize here that, despite the instability of the antitoxin, cells containing such an operon survive probably because the frequency of translation termination is increased after the stop-codon of the antitoxin (for example due to the overlap and/or frame-shift between the two genes) making the antitoxin protein more abundant than the toxin protein. Once a bacteria has received one or more copies of the system (through cell division or horizontal transfer), that cell is 'condemned' either not to loose the operon or to die (Fig. 1b) because absence of the operon would cause a reduction in the amount of antitoxin relative to the amount of toxin through degradation of the former (that cannot be replenished) prompting, in turn, the poison to exert its toxicity on an essential intracellular host target, such as the gyrase or RNA translation mechanisms [6,13,14], eventually causing cell death or cell-cycle arrest. Note that TA systems have been domesticated as biotechnological tools [15] for facilitating DNA engineering and protein production without the use of antibiotics [16].

Different TA systems have been described and classified in several families, according to the target of the toxin and/or the nature of the protease that degrades the antitoxin [17]. Recently, about 150 toxin genes have been separated into 4 groups on the basis of sequence or structure similarities and gene neighborhood criteria [18]: the "families" *relE/parE*, *mazF/kid/ccdB*, and *Doc*, as well as the family of proteins sharing a "PIN-domain". On the basis of phylogenetic analyses, these families have been suggested to be non-homologous [18], *i.e*., the TA systems would have appeared at least four times independently during evolution.

As known TA systems, identified on different plasmids, phages, and prokaryotic (including archaeal) genomes are all very small and potentially very divergent (TA systems originated from one or a few very old radiations), we hypothesize that many TA systems might not be annotated as such in the NCBI database. However, given that *(i) *TA-bearing plasmids with broad host range can be found in multiple bacterial species, and *(ii) *most systems exhibit the structural organization outlined above, we predicted that many more descendent systems than previously described should be detected across a wide range of prokaryotic genomes and plasmids. To detect these putative TA systems, we developed an algorithm, implemented into a computer program, *TAQ V1.0 *(for *"TA Query"*), that searches public databases on the basis of predefined similarity and TA-specific structural constraints. Our algorithm is complementary to that implemented in RASTA-Bacteria [19]. The latter first identifies sequences exhibiting conserved putative TA domains and then uses structural constrains to further restrict and score the resulting set of putative TA systems. Our approach, using a single starting query sequence for each of the ParE, Doc, and PIN families and two starting sequences for the MazF/CcdB family identified over 1,500 putative TA systems, of which many were unknown. These five groups of sequences are analyzed phylogenetically for a better classification and understanding of TA systems evolution.

## Results and Discussion

In order to produce a single set of homologous sequences per family, we performed one TAQ V1.0 search for each family of TA systems starting with a single poison (the ParE toxin from the *E. Coli *RK2 plasmid for the *relE/parE *family [20]; the MazF toxin from the delivery vector pIEF16S for the *mazF/kid/ccdB *family, [21]; the VapC toxin from *Leptospira interrogans *[serovar Lai str. 56601] for the PIN-domain family [18,22], and the Doc toxin from the Enterobacteria phage P1 for the *Doc *family, [23]). Since we did not find any CcdB sequence during the search using the MazF toxin as query (despite that CcdB and MazF are thought to belong to the same family), we also run one search using CcdB from the F plasmid as the starting sequence. In each case, the program TAQ V1.0 converged after 1 or 2 days of computation on a standard Intel Pentium 4 computer (2.0 GHz) and generated the results summarized in Table 1.

**Table 1:** Taxonomic distribution of the *in-silico *inferred toxins identified for the 5 TAQ runs.

Query sequences	*In silico *inferred toxins				Total *in silico *inferred toxins	Bad Poisons	Without antinodes poisons
				
	Bacteria	Archaea	Viruses	Artificial & Plasmids			
ParE	665	38	0	7	710	102	387
MazF	205	14	1	7	216	88	196
Doc	83	1	2	0	86	59	43
CcdB	20	0	0	2	22	67	85
VapC	717	143	0	0	860	121	87

Values represent the number of sequences in each category. The " *in silico *inferred toxins" are sequences that meet all the constraints implemented in TAQ whereas the "bad poisons" do not match the length constraints and the "without antidote poisons" meet the size constraints but do not exhibit an ORF of constrained size and position (see text for details). "Artificial" sequences are engineered sequences such as cloning vectors.

Although induced expression of the bacterial RelE toxin in yeast and in human cell lines indicated the broad potential activity of TA systems [24,25], none of the sequences that met all the sequence similarity and structural criteria defined in our algorithm are found in eukaryotic genomes. On the other hand, other categories include eukaryotic sequences: *e.g*., *Tetrahymena thermophyla *(Alveolata), *Debaryomyces hansenii *(Fungi), and *Dictyostelium discoideum *(Mycetozoa) in the ParE *"Bad poisons" *category, and *Cryptosporidium hominis *(Alveolata) in the ParE *"without antidote poisons" *category; *Aspergillus fumigatus *(Ascomycota), *Coccidioides immitis *(Ascomycota) *Macaca mulatta *(Mammalia), *Drosophila melanogaster *(Insecta), *Mus musculus *(Mammalia), *Homo sapiens *(Mammalia),*Gallus gallus *(Aves), *Rattus norvegicus *(Mammalia), *Pan troglodytes *(Mammalia), *Canis lupus *(Mammalia), *Bos taurus *(Mammalia) in the Doc *"Bad poisons" *category. Some of these sequences contain a domain (Fic domain in the *Drosophila *and *Mus *sequences; HYPE domain in other mammals and the chicken) that has been suggested to be homologous to the Doc domain [18].

**Figure 2:**
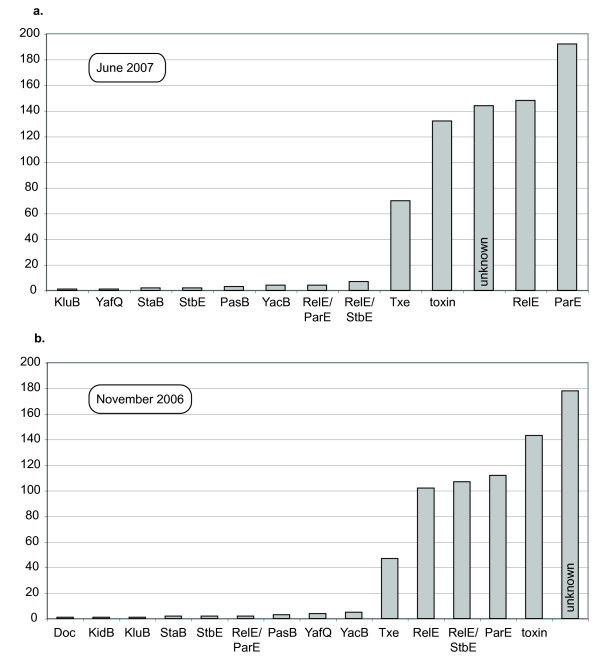
Assignment of the in-silico inferred toxins to functional categories (following the NCBI annotations) for ***(a *and *b) ***ParE runs performed at a 7 months interval.

Figure 2a shows the assignment of the ParE *"in-silico inferred toxins" *to functional categories. Note that this classification is simply based on the annotation of the corresponding sequences as provided by the NCBI database. The bulk of the sequences uncovered by our algorithm are annotated by the NCBI as "ParE", "RelE", "unknown", and "toxins". Note that annotation of the NCBI database is improving fast: the "unknown" category moved from the most frequent to the third most frequent category in 7 months (Fig. 2a&2b). Similarly, the erroneous annotation of 2 sequences as "Doc" and "KidB" disappeared in the most recent run. We suggest that, without accurate and extensive functional data (for example on the exact mechanism by which each poison exerts its action), a phylogenetic approach might generate the most appropriate and objective classification (*e.g*., because it is likely to be reasonably stable to the addition of new sequences). Note that one preliminary run of our program TAQ V1.0 with low BLAST stringency (*E-value *of 1 instead of 0.1) collected about 30 sequences annotated as belonging to the "merR" gene family, whose members are involved in metallic-ion metabolism [26]. Visual inspection of the run log files indicates that the low stringency of the similarity search generated a (probably inapt) hit on one merR protein, which, in turn (when used as query), collected additional members of the family. The merR genes happened to meet the structural constraints because they form operons with short genes arranged in tandem. Note that changing the *E-value *from 1 to 0.1 caused the loss of very few sequences of the category "unknown", suggesting that the merR family was the only significant artifact.

**Figure 3:**
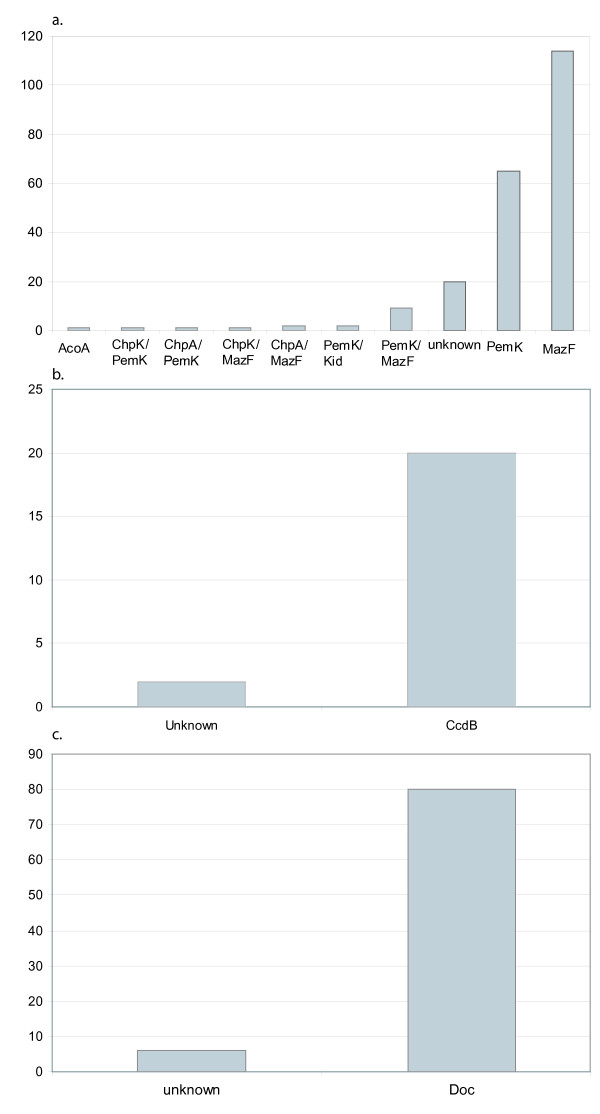
***(a) ***Assignment of the in-silico inferred toxins to functional categories (following the NCBI annotations) for the MazF run, ***(b) ***the ccdB run and ***(c) ***the Doc run.

Figure 3a shows the assignment of the 216 MazF *"in-silico inferred toxins" *to functional categories. Most sequences uncovered by our algorithm are annotated by the NCBI as "ChpA", "ChpK", "MazF", "PemK", "Kid", and "unknown". The frequency of the latter category is much smaller than in the run using ParE as query. Note that one hit in the MazF analysis is an acetyl-CoA acetyltransferase ("AcoA"), but close inspection of the NCBI file indicates that annotation was automatic (no functional experimental evidence) and we included this sequence in our phylogenetic analyses.

Figure 3b shows the assignment of the 22 CcdB *"in-silico inferred toxins" *to functional categories. Only two sequences are "unknown", all the others are CcdB annotated.

Figure 3c shows the assignment of the 86 Doc *"in-silico inferred toxins" *to functional categories: about 10% of the sequences are annotated as unknown, whereas all remaining sequences are annotated as "Doc".

The run that was started with a single member of the PIN family generated 1418 putative toxins, 544 bad poisons, and 611 without-antidote poisons. Among the 1418 putative toxins, 381 are known VapC toxins, 163 are unknown, and 556 are simply annotated as containing a PIN domain. However, as the PIN domain is not specific to TA systems, it is highly likely that the first step of our TAQ algorithm (i.e. the BLASTP search) returns many false positives (i.e., sequences that are not TA systems) that are not efficiently filtered out by the structural-constrain criteria. One example is the FlbT protein clearly involved in the flagellum biosynthesis [27]; note that one cannot exclude the possibility that some proteins regulating gene expression (e.g., the FlbT gene product may act as a negative regulator of the flagellin fljK gene expression [28]) originate from TA systems. Surprisingly, TAQ even identified known Phd antitoxins. Hence, to avoid comparing non-homologous sequences, we decided to decrease the *E-value *to 10^-3^. Under that setting, TAQ did not recover any FlbT, Phd, or any other sequence that can readily be identified as false positives.

**Figure 4:**
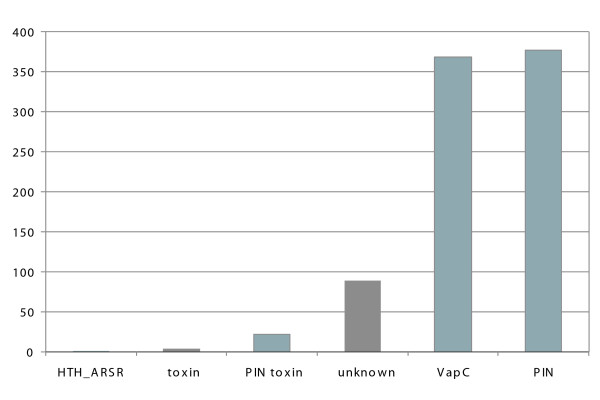
Assignment of the in-silico inferred toxins to functional categories (following the NCBI annotations) for the PIN run.

This second run generated 860 putative toxins, 121 bad poisons, and 87 without-antidote poisons. Among the 860 putative toxins, 368 are known VapC toxins, 89 are unknown, 25 are annotated as toxins and 377 as containing a PIN domain (Fig 4).

We compared the results of TAQ to those obtained by RASTA-Bacteria [19], a program that searches for putative TA systems in a specific organism but that restricts the BLAST search to known TA domains before applying additional structural constraints (ORF size, occurrence of an operon). RASTA-Bacteria finds many more putative TA elements (including isolated putative poisons or putative antidotes) than does TAQ because the latter uses more stringent size criteria (and constraint for the presence of both a toxin and an antitoxin genes) to minimize the risk of false positives that would seriously jeopardize the phylogenetic analyses. RASTA-Bacteria and TAQ have different objectives as the former attempts to score putative Toxin or Antitoxin elements whereas the latter attempts to generate a phylogeny among poison sequences that are very likely to belong to real TA systems (because they are all associated to a putative antidote and they all meet multiple size and localization criteria).

For a meaningful evolutionary analysis of the large sets of proteins recovered by TAQ, an additional criterion (e.g., the use of RASTA-Bacteria to test for the presence of an antitoxin domain in the putative antitoxin sequence) would be warranted. Unfortunately, RASTA-Bacteria does not allow, in its present form, to perform automated multiple searches using batch files.

### Ancestral sequences tree

After initially grouping sequences by query, we manually separated or merged groups for generating low ambiguity alignments. We used 35 groups for the ParE analysis, 12 groups for the MazF analysis, 7 groups for the Doc analysis, and 54 groups for the VapC analysis. Sequences within each group were then aligned and used to produce ML phylogenies. Then, the 35 inferred ML root sequences (MLRS) for the ParE analysis (12, 7, and 54 MLRS for the MazF, Doc and VapC analyses, respectively) were themselves aligned and analyzed phylogenetically (see Methods for details). The final trees and their branch support values are available through TIQ v1.0, a Java program, available at [29] (using the login "tiq" and password "yqgWrj.81"). TIQ allows browsing the trees, select branches, perform searches of NCBI annotation fields (such as sequence, taxa names, Global Identifiers (GI), etc.) and filter sequences according to the host taxonomy. The 22 CcdB sequences were simply incorporated into a single MrBayes analysis.

### Supertree

For the ParE analysis, after grouping hits on the basis of the query sequence(s) that generated them, we obtained 710 groups of which many were highly redundant. Using the algorithm described in the Methods section, we reduced that number to 111 partially-overlapping groups that collectively contain all putative TA systems uncovered here. We then inferred the ML phylogenetic relationships among sequences within each group, generating 111 trees that are partially overlapping in terms of the included sequence. The overlapping trees were then used as input for "supertree" inference (see Methods section). The strict consensus among the 4 best supertrees (score 310.49) is available through TIQ v1.0 (see above). Similarly, for the MazF analysis, the initial 227 highly redundant groups were reduced to 76 partially-overlapping groups that collectively contain all uncovered putative TA systems. The single best supertree (score 157.57) is available through TIQ v1.0. For the Doc analysis, the initial 86 highly redundant groups were reduced to 9 partially-overlapping groups that collectively contain all uncovered putative TA systems. The strict consensus among the 17 best supertrees (score 11.72) is available through the java program TIQ v1.0. Finally, for the VapC analysis, the initial 860 highly redundant groups were reduced to 208 partially-overlapping groups that collectively contain all uncovered putative TA systems. The 50% majority-rule consensus among the 115 best supertrees (score 246.91) with a cut-off value of 50% is available through the java program TIQ v1.0. No supertree analysis was run for the 22 CcdB sequences given the small size of that dataset.

### Consensus among methods

**Figure 5:**
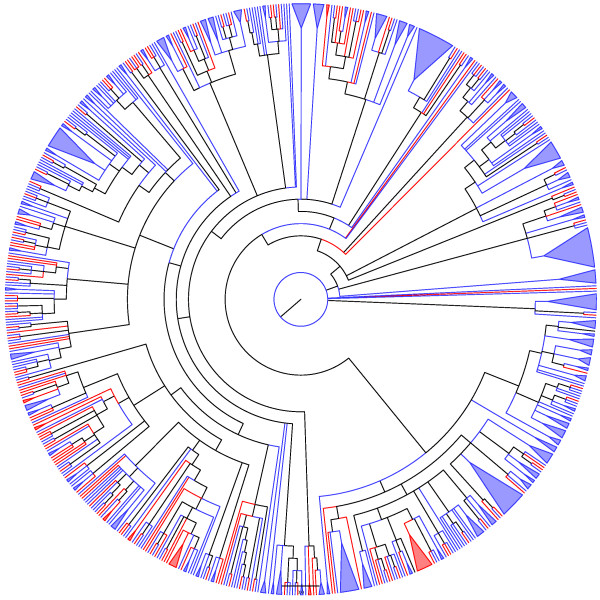
**Adams consensus among tree generated through the two heuristics used here ("ancestral tree" and "supertree", see text for details) for the ParE family.** Blue and red lineages represent known and unknown TA systems, respectively.

**Figure 6:**
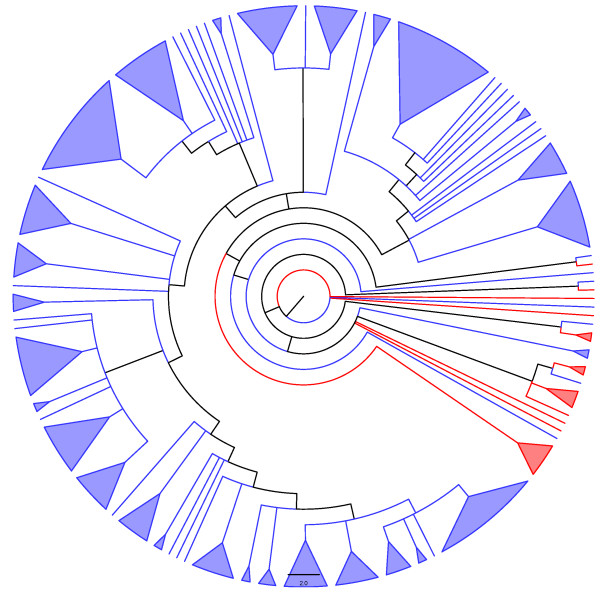
**Adams consensus among tree generated through the two heuristics used here ("ancestral tree" and "supertree", see text for details) for the MazF family.** Blue and red lineages represent known and unknown TA systems, respectively.

**Figure 7:**
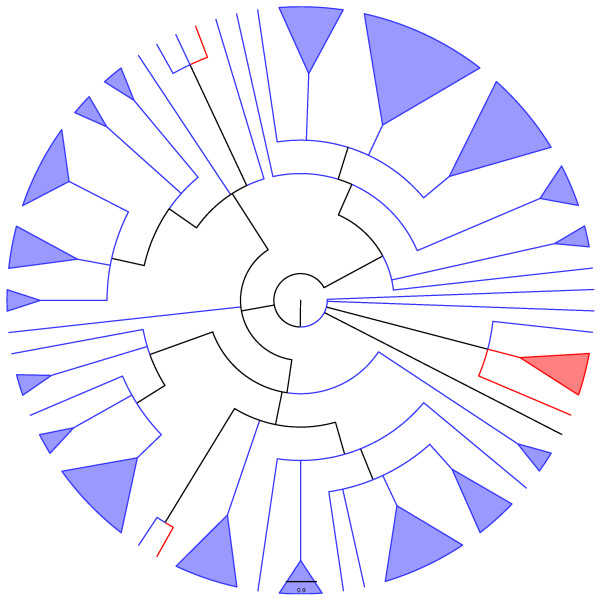
**Adams consensus among tree generated through the two heuristics used here ("ancestral tree" and "supertree", see text for details) for the Doc family.** Blue and red lineages represent known and unknown TA systems, respectively.

**Figure 8:**
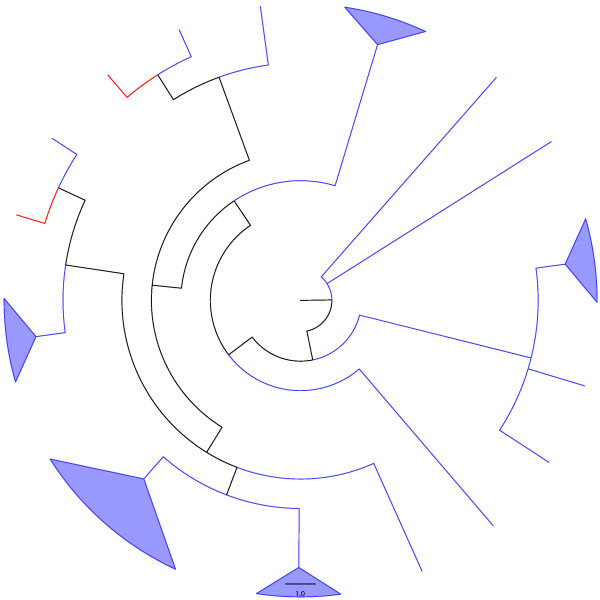
**MrBayes tree of the ccdB family.** Blue and red lineages represent known and unknown TA systems, respectively.

**Figure 9:**
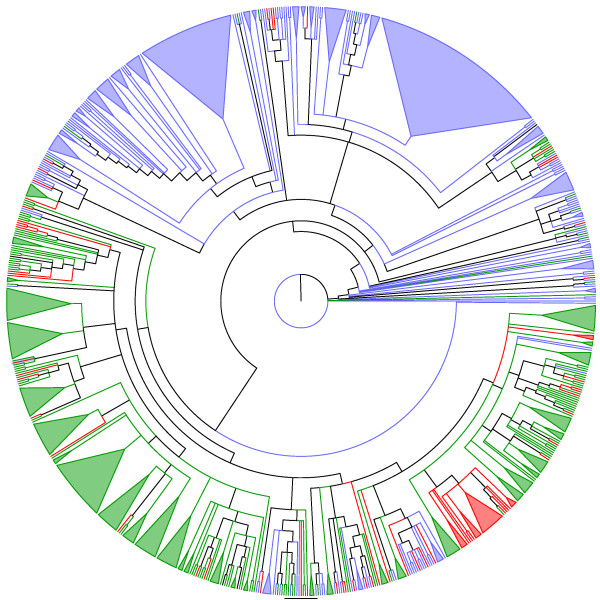
**Adams consensus among tree generated through the two heuristics used here ("ancestral tree" and "supertree", see text for details) for the VapC family.** Blue and red lineages represent known and unknown TA systems, respectively. Green lineages represent sequences annotated as "containing a PIN domain".

Given *(i) *the very large divergence among the very short poison sequences from TA systems (even within each of the major families: ParE, MazF, Doc and VapC), *(ii) *the heuristic nature of the inference methods used here (see below), and *(iii) *that ML models used are only approximations of the real substitution process across the billions of years of TA systems history, a high degree of uncertainty is very likely attached to the phylogeny analyses performed here. However, given that the two heuristics used are quite different (*i.e*., either inferring relationships among reconstructed most likely ancestral sequences of predefined groups of poison sequences or using a supertree approach among a set of overlapping trees), a consensus between the two resulting phylogenies might highlight the most robust portions of these trees. Hence, for each major family, we combined the trees generated by the two approaches into an Adams consensus tree (Fig. 5, 6, 7 and 9). An Adams consensus [30] typically preserves more structure than a strict consensus by displaying "nestings", rather than clades, shared among trees (given two sets of leaves, A and B, and a tree T, set A nests inside set B if *(i) *A is a subset of B, and *(ii) *the leaves in set A have a more recent common ancestor in T than do the leaves in set B). In other words, one group is said to nest within a larger group if the most recent common ancestor of the smaller group is a descendant of the most recent common ancestor of the larger group, which needs not require monophyly of either group. The consensus trees, presented in Figures 5, 6, 7 and 9 are also available (for browsing and searching) through the java program TIQ v1.0 (see above). The CcdB tree (Figure 8) is also available through TIQ.

### Origin of the TA systems

Very little objective data is available to shed light on the origin of the TA systems. Considering the structural relative similarity (*i.e*., the criteria implemented in our software TAQ V1.0, see Methods section) among known systems, Gerdes [17] suggested that they all share a common ancestor. However, the evolutionary relationships and functional (dis)similarities among TA families are unclear. For example, the similarities between, on one hand, toxin sequences from the RelE family and, on the other hand, those from the ParE family (see refs [8,31] and analyses above) demonstrate that the two families are homologous (*i.e*., they share a common ancestor). However, RelE and ParE proteins are thought to exert their toxic activity on different targets: mRNA cleavage for RelE and DNA gyrase for ParE [7]. Similarly, MazF and CcdB proteins are thought to be homologous because they share the same basic tertiary structure [31,32]. Finally, Schmidt *et al*. [33] described an "hybrid" TA system, *i.e*., whose antidote sequence is similar to the MazE antitoxin, whereas its toxin sequence is similar to RelE, providing a putative evolutionary link between the RelE/ParE and MazF/CcdB superfamilies.

Conversely, other scholars suggested that TA systems evolved several times independently. For example, on the basis of protein domains and "gene-neighborhood analysis", Anantharaman and Aravind [18] proposed that RelE/ParE, MazF/CcdB, Doc, and PIN form different TA superfamilies that have been assembled more than once during the evolution, from a limited pool of protein domains.

To test for the possible homology among TA superfamilies, we run TAQ using PSI-BLAST criteria (e-value of 10.0 and PSI-BLAST threshold of 0.1, 10 iterations) more permissive than in a classical TAQ run (see Methods). Using ParE as input, we found 4ζ toxins from the ω -ε -ζ system, as well as a sequence containing the PIN domain (previously detected in some VapC toxins [18] of the VapBC system [22]). This result suggests possible homologies among ParE/RelE, ζ, and VapC families, and the rapid accumulation of new prokaryotic genome sequences might bridge additional putative phylogenetic gaps among TA families. Moreover, our first run of TAQ with a VapC toxin as initial query retrieved some Phd antitoxins (see above).

We also input 303 sequences of putative toxins (139 referred as "toxins" and 164 referred as "unknown") uncovered by TAQ V1.0 into RASTA-Bacteria [19]. When RASTA-Bacteria gave more than one possible domain per sequence, we only considered the domain with the highest score. Strikingly, seven of our putative poisons belonging to the RelE superfamily (Figure 5) were assigned non-RelE domains (a Doc, PemK, CcdB, VapC, and PIN domain for 3, 1, 1, 1, and 1 sequences, respectively) by RASTA-Bacteria. Although these results exhibit low scores, they might prove to be the first objective link, in terms of homology, among TA superfamilies. *In-vivo *experiments, identifying the nature of the domains by which these proteins exert their putative toxicity, would probably shed light on this exciting hypothesis (see below).

## Conclusion

**Figure 10:**
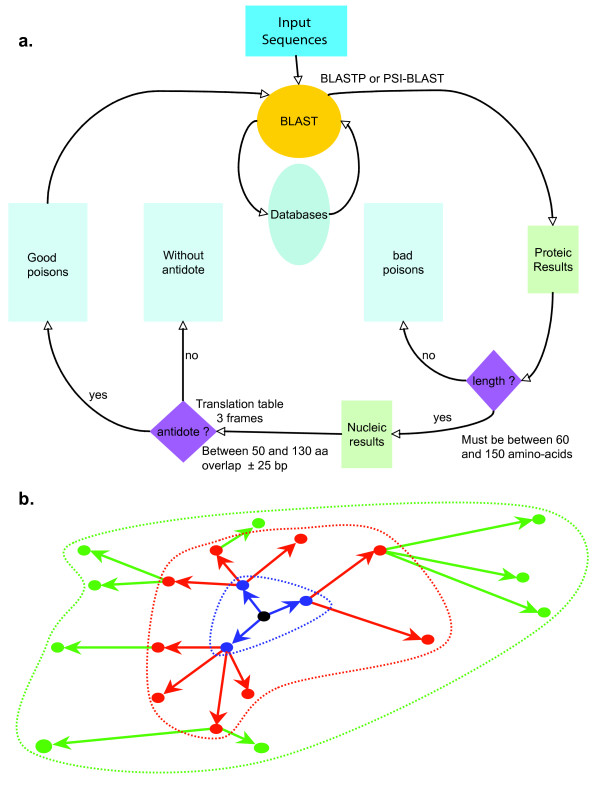
***(a) ***The algorithm implemented in the program TAQ V1.0 for the detection of putative TA systems. See text for details. ***(b) ***Dynamic of solution finding by the TAQ V1.0 program. The central black dot stands for the initial input sequence, and the colored dots for the "good poisons". During the first BLAST (in blue) new poisons are discovered (blue dots) and used as queries for new BLASTs (in red then in green). When the total solution space (dotted-line delimited) does not grow anymore, convergence is reached and the search stops.

Figure 5 indicates that among the *"in-silico inferred ParE toxins"*, *i.e*., the new putative TA systems (red lineages) identified by our search algorithm (Fig. 10) are widely distributed into the phylogenetic tree of the ParE family. Conversely, the new putative TA systems uncovered here for the MazF, Doc, and VapC families are more restricted in their phylogenetic localization (Fig. 6, 7 and 9). However, for the MazF family, the new putative TA systems are particularly ancient (hence, divergent) in the phylogeny of the family, emphasizing the efficiency of our algorithm for identifying new TA systems.

All analyses presented here include exclusively sequences that met all similarity and structural constraints implemented in TAQ V1.0, *i.e*., the "*in-silico *inferred toxin" sequences. However, many additional hits meet the similarity criterion but fail to meet either the size criterion or the presence of a putative antidote (*"bad toxins" *or *"without-antidote putative toxins" *sequences, respectively). It would be particularly interesting to extend the phylogenetic analyses performed here to these additional sequences, especially for those found in eukaryotic genomes, to assess their likelihood to represent functional or degenerated poison genes.

Putative toxins particularly divergent in the phylogenies presented in figure 5, to 9, as well as those making the possible link between different TA superfamilies (see above), must be tested *in vivo *to *(i) *authenticate their toxic nature, *(ii) *identify their mode of action, and/or *(iii) *possibly confirm their hybrid character (*i.e*., with the toxic mode of action from family A, and the antitoxin structure of family B). Finally, it would be worth investigating which of the two proteases known to degrade antitoxins are active against the antitoxins of the tested putative TA systems.

## Methods

### Search for TA homologs

Given the small size of and the very large divergence among TA systems, we assumed that many might not be annotated as such in the NCBI database. Hence, we developed an algorithm and implemented into a computer program, TAQ V1.0, for the detection of putative TA systems (Figure 10a). The program starts with an input file that contains one or more amino-acid poison sequence(s) in FASTA format. The first sequence in the list is used for a sequence similarity search against a local copy of the "nr" public database (non-redundant protein sequence database; see [34] for additional information). The local alignment algorithm used is BLASTP V2.2.9 or PSI-BLAST V2.2.9 (Position-Specific Iterated – BLAST). The latter is a method that automatically combines statistically-significant alignments produced by BLAST [35] into a position-specific score matrix; the database is then searched using this matrix [36,37]. PSI-BLAST was used with an *e-value *threshold (E) of 0.1 (*i.e*., 0.1 expected random hit with the same score, given the sizes of the query and database) and 4 iterations. All other parameters were set as default.

The next step of the algorithm consists into using structural parameters to check if BLAST hits are likely to belong to TA systems. To this end, TAQ V1.0 uses the annotated NCBI file (listing all hits) generated by BLAST to identify, for each amino-acid sequence hit, the corresponding genomic region and nucleotide sequence. Sequences are then filtered according to the following successive criteria (Figure 10a): *(i) *the length of the putative toxin (*L*_*pT*_) must be 60aa <*L*_*pT *_< 150aa, a range encompassing the length distribution of all known toxin genes plus 40 aa; *(ii) *an additional ORF (putative antitoxin) of length 40aa <*L*_*pA *_< 150aa must be found (using the appropriate translation table and the three phases) in the 410 bp interval downstream of the putative toxin ORF; *(iii) *the separation or overlap between the putative antitoxin and putative toxin ORFs (*i.e*., between the stop codon of the former and the start codon of the latter) must be of maximum 30 bp. Only the sequences meeting all the above conditions are stored as "*in-silico *inferred toxins" (and the corresponding antitoxins are stored in a separate database) for further analyses. For each run, we assume that all sequences retained are *(i) *TA systems (active or inactive), and *(ii) *homologous (*i.e*., share a common ancestor). Every new "*in-silico *inferred toxin" sequence is automatically fed as a query into the TAQ V1.0 software until it converges (*i.e*., until no new sequence is found, Figure 10b). BLAST hits that do not meet the size criterion are stored in a *"bad toxin" *database, whereas BLAST hits that meet the size criterion but cannot be associated to a putative antitoxin are stored in a *"without-antidote putative toxins" *database. It is indeed conceivable that real toxin genes can lose their activity, hence, their associated antitoxin, during evolution.

In order to produce a single set of homologous sequences, we performed one TAQ V1.0 search for each family of TA systems starting with a single poison (the parE toxin from the *E. Coli *RK2 plasmid for the *relE/parE *family, the MazF toxin from the delivery vector pIEF16S for the *mazF/kid/ccdB *family, the Doc toxin from the Enterobacteria phage P1 for the *Doc *family, and the VapC toxin from *Leptospira interrogans *[serovar Lai str. 56601] for the PIN domain proteins). Since we did not find any CcdB sequence during the search using the MazF toxin as query (despite that CcdB and MazF are thought to belong to the same family), we also run one search using CcdB from the F plasmid as the starting sequence.

### Alignment and phylogenetic analyses

**Figure 11:**
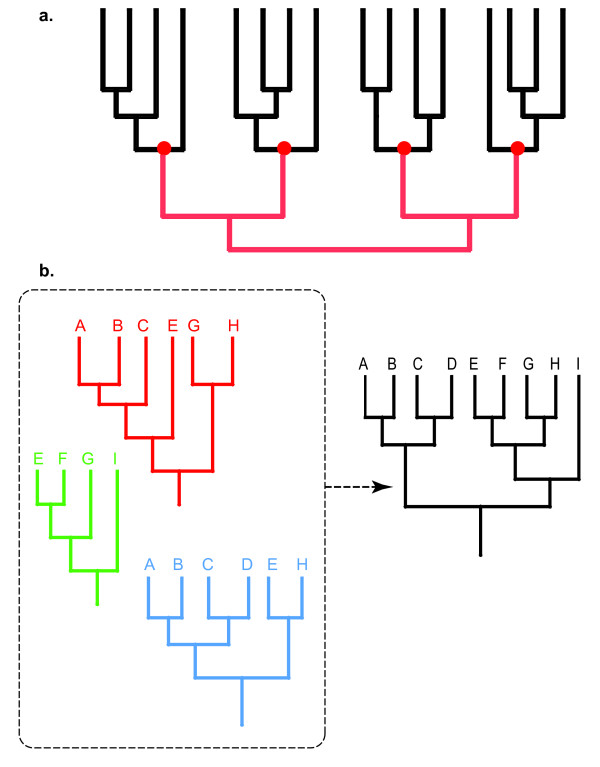
***(a) ***Schematic of the "ancestral sequence tree" method used for inferring the phylogeny among putative toxins sequences uncovered by TAQ V1.0. In this hypothetical example, the sequences are separated into 4 groups. Black edges represent the ML phylogenies within each of the 4 considered groups. The red dots stand for the inferred ancestral sequence of each group. The red edges define the phylogeny among the inferred ancestral sequences. ***(b) ***Schematic of the second heuristic ("super tree" approach) used for inferring the phylogeny among putative toxins sequences uncovered by TAQ V1.0. In this hypothetical example, the sequences are separated into 3 groups. The red, green, and blue trees represent the ML phylogenies within each of the 3 considered groups. These source trees are then used collectively for generating the best super tree, *i.e*., the tree including all sequences that is most compatible with all source trees (see Methods section for details).

Given the difficulties to analyze sequence matrices including a large number of short and highly divergent sequences, we used several criteria to separate them into groups. First, we grouped all sequences found with the same query. Large groups were sometimes very difficult to align and were manually separated into sub-groups until alignment became much less ambiguous. On the contrary, very small groups (< 6 sequences) were manually combined with a larger group with which they aligned best. Final alignment among aa sequences within each group was generated with PROALIGN V0.4 (available at [38]), a program implementing a method for multiple sequence alignment that combines an HMM approach, a progressive alignment algorithm, and a probabilistic evolution model describing the character substitution process [39]. This method allows the assignment of a posterior probability to each aligned position at each node of the guide tree and has been demonstrated to perform particularly well in benchmarks comparing different approaches [40]. Each alignment was used to produce a maximum likelihood (ML) phylogeny (see below), and the most likely ancestral (root) sequence (MLRS) was estimated using software GASP [41], with mid-point rooting. MLRS (one per alignment) were then themselves aligned using PROALIGN and their ML phylogenetic relationships were inferred (see below). The final tree was built by replacing MLRS, on the tree among MLRS, by the corresponding initial trees (Figure 11a).

ML phylogeny inference was carried out with the Bayesian approach [42] implemented in MRBAYES 3.1.2 [43,44] with the *"mixed protein model"*. The Markov chain Monte Carlo search was run with 6 chains for 10^6 ^millions generations and an initial temperature of 0.1, with trees sampled every 100 generations (the first 2,500 trees were discarded as "burnin"). Hence, Bayesian posterior probabilities were estimated as the majority-rule consensus tree among the 7,500 last sampled trees.

As an alternative to the above-mentioned approach (performing phylogenetic analysis among MLRS inferred from sub-groups of sequences), we also applied the supertree method [45,46]. To this end, we requested our program TAQ V1.0 to output the query sequence(s) that generated each hit. Different poison sequences were grouped when they had been generated by the same query sequence. As different queries can generate the same hit, the groups are partially overlapping. As many of these groups are highly redundant (some group are even fully included into others), we reduced the number of groups using the following rules. First, groups of less than 3 sequences are discarded. Second, groups are sorted by decreasing size; groups 2 to N are compared each with group 1 (the largest group); any group sharing more than 2 sequences with group 1 are discarded and group 1 is set aside; the procedure is then iterated using the remaining groups. Once the two last groups have been compared, we checked that all toxin sequences in the initial list were included in the union of all groups set aside. Note that for the MazF family, the maximum allowed overlap between groups was raised from 2 to 3. We then inferred the phylogenetic relationships among sequences (using MRBAYES 3.1.2 with the *"mixed protein model"*) within each of the N groups, generating N trees that are partially overlapping in terms of the included sequence. The overlapping trees were then used as input for "supertree" inference (Fig. 11b) using CLANN v 3.0.3d [47]. This program implements 5 different methods of supertree reconstruction, of which only the *"Most Similar Supertree" *approach allowed analysis of the RelE and VapC datasets in practical computing time. This hill-climbing heuristics consists into generating a supertree topology T_*i *_(*i.e*., containing all taxa as leaves) at iteration *i *by performing a random branch swapping (following the SPR algorithm, [48]) on the topology T_*i*-1 _generated at iteration *i*-1. T_*i *_is accepted as a new starting topology if its score (evaluated under an optimality criterion described below) is better than that of tree T_*i*-1_, otherwise it is rejected and a new branch swapping is performed on T_*i*-1_. The score of a proposed supertree topology is evaluated as follows. First a taxa distance matrix is computed for each of the N source trees: the distance between a pair of taxa is the number of nodes that separate them in the source tree. Second, each source tree topology is compared to the proposed supertree topology: for each comparison, the supertree topology is pruned of all the taxa that are not present in the source tree and the supertree distance matrix is computed. Third, the score of the supertree topology relative to a given source tree is computed as the absolute difference between the distance matrix of the latter and that of the corresponding pruned supertree. Finally, the full score of the proposed supertree topology is computed as the normalized sum of all scores. For each major toxin family (ParE, MazF, and Doc) we performed 10 CLANN (v 3.0.3d) runs and kept all equally best trees. For the VapC family, we performed 5 runs (each using a starting trees generated using various heuristics since CLANN did not manage to build a starting tree from our set of trees) and kept all equally best trees.

## Authors' contributions

MCM and CS conceived the study. JG wrote the programs TAQ v1.0 and TIQ, and performed the phylogenetic analyses. MCM and JG wrote the manuscript. All authors commented on the draft manuscript and approved the final manuscript.
